# Deferasirox, an Iron-Chelating Agent, Improves Testicular Morphometric and Sperm Functional Parameters in a Rat Model of Varicocele

**DOI:** 10.1155/2021/6698482

**Published:** 2021-04-08

**Authors:** M. Rahmani, M. Tavalaee, M. Hosseini, A. Eskandari, E. Shaygannia, N. Sadeghi, M. N. Nazem, P. Gharagozloo, J. R. Drevet, M. H. Nasr-Esfahani

**Affiliations:** ^1^Department of Animal Biotechnology, Reproductive Biomedicine Research Center, Royan Institute for Biotechnology, ACECR, Isfahan, Iran; ^2^Department of Basic Science, School of Veterinary Medicine, Shahid Bahonar University of Kerman, Kerman, Iran; ^3^CellOxess LLC, 830 Bear Tavern Road, Ewing NJ 08628, USA; ^4^GReD Institute, Faculty of Medicine, INSERM-CNRS-Université Clermont Auvergne, France; ^5^Isfahan Fertility and Infertility Center, Isfahan, Iran

## Abstract

Varicocele is characterized by testicular dysfunction that originates from hyperthermia and hypoxia, leading to defects in testicular tissue and altered spermatozoa structure and function. The varicocele testis is characterized by the presence of intracellular iron deposits that contribute to the associated oxidative stress. Therefore, we tested the hypothesis that administration of an iron-chelating agent, such as deferasirox (DFX), could potentially mitigate the consequences of varicocele on testicular tissue and spermatozoa. Using a well-established rat model of varicocele (VCL), we show that treatment with DFX partially improved the structure and function of the testis and spermatozoa. In particular, sperm motility was markedly restored whereas abnormal sperm morphology was only partially improved. No significant improvement in sperm count was observed that could be associated with the proapoptotic response observed following iron chelation treatment. No reduction in oxidative damage to spermatozoa was observed since lipid peroxidation and DNA integrity were not modified. This was suggested to be a result of increased oxidative stress. Finally, we also saw no indication of attenuation of the endoplasmic reticulum/unfolded protein (ER/UPR) stress response that we recently found associated with the VCL testis in rats.

## 1. Introduction

Dilatation of the testicular pampiniform plexus associated with spermatic vein valve disorder is commonly referred to as varicocele (VCL) [1]. Epidemiological data indicate that VCL is present in 15–20% of the male population and is associated with reduced spermatozoa quality. Varicocele has a multifaceted etiology mainly related to increased testicular temperature and oxidative stress [2]. Although various molecular pathways are thought to be associated with VCL, including hormonal disorders, hypoxia, germ cell apoptosis, and disruption of the testis blood testicular barrier [1, 2], more research is needed to further our molecular understanding of this syndrome and how to improve patient treatment.

One logical approach to reduce the detrimental impact of VCL on testis structure and function would be to try to reduce the associated oxidative stress. We propose that control of iron homeostasis in the VCL testis could be a pertinent approach. Iron is one of the essential nutrients necessary for spermatogenesis. It is transferred to the testis through the circulation and is stored in testicular cells as ferritin, transferrin, and other storage proteins that comprise the “iron shuttle protein system” [3]. Iron imbalance has been shown to act as a double-edged sword in the reproductive tract [4] as iron deficiency hinders spermatogenesis, while excessive iron deposition can induce sterility. In VCL, it was reported that free iron is deposited in the testicles due to cell lysis following hyperthermia [5]. In addition, it is known that free radicals created by iron accumulation attack iron-containing cells and proteins, resulting in the release and deposition of iron in tissues [6–8]. In this respect, several studies have clearly shown an increase in testicular iron deposits in infertile patients with VCL as well as in rats with induced VCL [5, 9–11]. Iron is involved in the formation of the most toxic form of oxygenated radicals, the hydroxyl radical, following the classical Fenton and Haber–Weiss reactions [12]. As such, it is a major contributor to the oxidative stress that accompanies VCL. In addition to the testicular and spermatozoa oxidative damage that accompanies VCL, we recently used an induced model of VCL in a rat model to demonstrate that the endoplasmic reticulum/unfolded protein (UPR/ER) stress response [13, 14] is part of the VCL testis response. We showed that the classical mediators of the UPR/ER stress response (mainly the IRE1 pathway) were involved [15] confirming earlier reported data [16, 17]. The VCL testis ER response was shown to lead to germ cell apoptosis that may partially account for reduced spermatogenesis and sperm quality in VCL rats.

Deferasirox (DFX) is an orally absorbed iron chelator. It is a lipophilic molecule with a strong affinity for ferric iron (Fe^3+^) [18, 19] and has a bioavailability of approximately 70% with a long half-life facilitating its use (single daily oral administration). Two molecules of DFX can form a complex with ferric iron (Fe-[DFX]), to reduce iron overload in the body and prevent further iron deposition [18]. In this respect, Miao et al. (2020) demonstrated that DFX can prevent the Fenton reaction and the associated overproduction of reactive oxygen species (ROS) [20]. Our approach to investigate the effect of DFX in the VCL rat model is based on previous studies showing excess iron in the male reproductive tissue of infertile individuals and, in particular, VCL patients [9–11]. As a catalyst, iron plays a central role in the Fenton reaction that accompanies oxidative bursts, with lipid peroxidation as the main consequence of ROS overproduction [4, 21]. In addition, ROS, in particular H_2_O_2_, damages iron-containing proteins leading to the release of iron into tissues [4, 6–8]. Since VCL is significantly associated with increased ROS and oxidative stress [22], we wanted to test whether a molecule such as DFX could reduce the deleterious effects of VCL, particularly with regard to the testicular ER/UPR response induced by VCL and its associated prooxidative and proapoptotic facets.

## 2. Materials and Methods

The mouse phospho-JNK monoclonal antibody (#9255) and the mouse CHOP monoclonal antibody (#2895) were supplied by Cell Signaling Technology (Danvers, MA, USA). The anti-GPX4 monoclonal antibody (#ab125066, EPNCIR144) was provided by Abcam (Cambridge, MA, USA). Goat-rabbit horseradish peroxidase (HRP) IgG (#Sc-2301), goat-mouse HRP IgG (#P0447), and mouse anti-*β*-actin (#A2228) antibodies were purchased from Santa Cruz (CA, USA), Dako (Carpinteria, CA, USA), and Sigma (St. Louis, MO, USA), respectively. The dyes acridine orange (AO) and chromomycin A3 (CMA3) were obtained from Sigma (St. Louis, MO, USA). Deferasirox (DFX) dispersible tablets (OSVERAL®, each tablet containing 250 mg DFX) were purchased from OSVE Pharmaceutical Co. (Tehran, Iran).

### 2.1. Experimental Design

**Figure 1:**
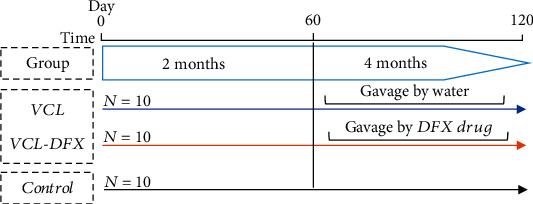
Flowchart of the different experimental sets (*n* = 10 each). VCL was induced surgically on day 0 in groups VCL and VCL-DFX. After 2 months, the VCL animals were given water while the VCL-DFX animals were given DFX (2 mg/kg) for 2 more months. Only one group of rats was used as controls to avoid excessive animal use as we demonstrated earlier that there was no difference between control and sham-operated animals for the parameters monitored [24].

This study was approved by the ethics committee of the Royan Institute (IR. ACECR.ROYAN.REC.1397.224). Thirty male Wistar rats (7–8 weeks, 180–220 grams) were obtained from the Royan Institute of Biotechnology (Isfahan, Iran). They were fed and maintained in accordance with the guidelines of the Ethics Committee of the Institute for Laboratory Animal Research. As shown in Figure 1, the rats were divided into three groups: the VCL group (*n* = 10), in which rats were administered water for 8 weeks beginning two months after surgical induction of VCL; the VCL-DFX group (*n* = 10), in which rats were administered 2 mg/kg DFX by gavage in place of water; and a final control group (*n* = 10), in which rats did not undergo any surgery or gavage. The DFX dose was defined on the basis of human doses converted for use in a rat model [19, 23]. Sham-operated animals were not included in this experiment as previous data have shown that there were no differences with control animals for the evaluated parameters [24]. A DFX alone group of animals was not included as DFX was only used to counteract iron overload induced by VCL and was never intended to be administered alone.

All surgeries were performed under sterile conditions. In brief, each rat was anaesthetized with an intraperitoneal injection of ketamine (75 mg/kg) and xylazine (2.5 mg/kg). After shaving the abdominal region, a polyvidone-iodine antiseptic was administered to the skin and an incision was made from the midline of the abdomen (xyphoid) to around the pubis in order to visualize the left renal vein, the left kidney, and the adrenal vein. Next, the inferior vena cava and the left renal vein were located by careful dissection. A 4-0 silk thread was placed and tied on the left renal vein near the branch of the inferior vena cava. The operation was carried out according to the protocol of Gholirad et al. [5]. It should be noted that this study is a continuation of our recent study published by Hosseini et al. [15] and that our animal model of VCL was verified on the basis of reduced semen parameters, histological findings, and functional semen tests [15, 25, 26].

### 2.2. Tissue and Spermatozoa Collection

All groups were sacrificed after 4 months. The final weight of each rat was recorded. The left testicular volume was determined according to Archimedes' principle by submerging the testicle in water. The left testicle was then divided into three portions: two portions were fixed in Bouin solution for histopathological evaluation and determination of iron content. The remaining portion was exposed to nitrogen vapour and then stored at -70°C prior to molecular analyses (protein and gene expression). The left cauda epididymides were recovered for the evaluation of sperm function. The tissues were dilacerated and incubated for 30 min at 37°C in VitaSperm medium (Inoclon, Iran). The following sperm parameters were evaluated: motility, concentration, morphology, nuclear DNA damage, membrane lipid peroxidation, and histone content.

### 2.3. Histological and Iron Assessment of Testicular Tissues

The fixed tissues were embedded in paraffin and sectioned (10 *μ*m). Sections were dewaxed, rehydrated, and stained with haematoxylin. The mean percentage of spermatogenesis (spermatogenesis index (SI)), meiotic index (MI), and Johnson's score in the seminiferous tubules were evaluated using an Olympus microscope (×40 magnification) and Dino Capture 2 imaging software (AnMo Electronics Corp., Torrance, CA, USA). For SI evaluation, the percentage of seminal tubules containing spermatozoa was defined [26]. For MI, the ratio between the number of round spermatids and primary spermatocytes was evaluated [27]. The degree of histopathological damage in tissue sections was determined according to Johnson's classification score [28]. In short, 100 different seminiferous tubules were randomly evaluated and each tubule was awarded a score between zero and ten. Finally, Johnson's total score was expressed as a percentage.

The free iron content was measured by the Prussian blue staining method (Perl's staining), which evaluates the Fe^3+^ (ferric iron) content. In brief, sections of dewaxed tissue were treated with a mixed solution (2% hydrochloric acid and 2% potassium ferrocyanide, ratio 1 : 1, *v* : *v*) for 20 min at room temperature. The sections were then washed with tap water and treated with rapid red (NovaUltra; IHC WORLD, LLC, Woodstock, MD, USA) for 5 min. Finally, the sections were dehydrated with successive washes in water, alcohol, and xylene [5, 29–32]. The images were captured using an Olympus BX51 microscope (×100 magnification), and the number of seminiferous tubules containing Fe^3+^ deposits was evaluated using ImageJ software.

### 2.4. Sperm Mobility, Concentration, Morphology, and Lipid Peroxidation

To determine sperm mobility, 10 *μ*l of the sperm suspensions extracted from the cauda epididymides was placed on a slide and then evaluated under a light microscope (CX31 OLYMPUS; ×40 magnification). Two hundred spermatozoa were observed from random microscopic fields, and mean motility was recorded. A Makler chamber was used to determine sperm concentration, which was presented as millions of cells *per* ml.

Sperm morphology was evaluated by staining 20 *μ*l of washed sperm with 40 *μ*l eosin at room temperature. After 5 min, 60 *μ*l of nigrosine dye was added. Smears were prepared with 20 *μ*l of the preparation [15]. Two hundred spermatozoa on each slide were randomly evaluated on an optical microscope (CX31 OLYMPUS, magnification ×100), and the total percentage of abnormal forms was determined.

The BODIPY® 581/591 C11 test was used to assess sperm membrane lipid peroxidation. The procedure was based on the protocol described by Aitken et al. [21]: two million washed spermatozoa were exposed to the BODIPY C11 probe at a final concentration of 5 mM/ml for 30 min at 37°C. A positive control was performed for each sample by adding H_2_O_2_ to the sample. The samples were then washed twice with PBS 1x to remove the unbound BODIPY C11 probe (500 g for 5 min). Finally, a flow cytometer FACSCalibur (Becton Dickinson, San Jose, CA, USA) was used to assess lipid peroxidation. The percentage of BODIPY-positive spermatozoa was recorded for each sample.

### 2.5. Sperm DNA Damage and Nuclear Integrity

The acridine orange (AO) test, which uses a cationic fluorescent dye, was performed to detect damage to sperm DNA. In brief, a smear of washed sperm was prepared on a slide and fixed with Carnoy's solution at 4°C. The slides were then stained with AO in citrate-phosphate buffer (80 ml 0.1 M citric acid+5 ml 0.3 M NaH_2_PO_4_, pH 2.5). The slides were washed with PBS 1x. Two hundred sperm were counted on each slide using a fluorescent microscope (B-383LD2 OPTIKA). Spermatozoa with a red or orange nucleus (see supplementary figure 1) were considered to show DNA damage [25].

The aniline blue (AB) test was used to assess the level of compaction of the spermatic nucleus. Aniline blue is an acid dye that interacts with lysine-rich histones. In brief, washed sperm cells were spread on slides, and after drying at room temperature, the slides were fixed with 3% glutaraldehyde [25]. Slides were stained with aniline blue (5% aqueous) in 4% ethanoic acid (5 min) and then washed with PBS 1x. A light microscope (×100 magnification, CX31 OLYMPUS, Japan) was used to randomly count 200 sperm cells in different fields for each slide. The percentage of spermatozoa with dark blue nuclei was noted and considered to be sperm cells exhibiting abnormal chromatin packaging (see supplementary figure 1).

Chromomycin A3 (CMA3) was used to evaluate the sperm nuclear protamine content. Washed spermatozoa were fixed with Carnoy's solution (methyl alcohol : ethanoic acid (3 : 1; *v* : *v*)) and stored at 4°C for 5 min. Then, 20 *μ*l of fixed spermatozoa solution was spread on slides and left to dry at room temperature. The slides were stained for 90 min with 150 *μ*l CMA3 solution (0.25 mg/ml CMA3 (St. Louis, MO, USA) in McIlvaine buffer containing 7 ml 0.1 M citric acid+33 ml 0.2 M Na_2_HPO_4_7H_2_O, pH 7.0, 10 mmol/l MgCl_2_). The slides were then rinsed in PBS 1x and covered with a cover slip. A fluorescent microscope (OPTIKA, CX31 OLYMPUS, Japan) was used to randomly count 200 spermatozoa in different fields. The percentage of spermatozoa with CMA3-labelled nuclei was noted (×100 magnification).

### 2.6. Isolation of Testicular RNA and qRT-PCR

In brief, 50 mg of tissue from each testicle was homogenized with 1 ml of TRIZOL (Invitrogen Co., USA) for 5 min at 25°C. The nucleic acids were then extracted by centrifugation according to the supplier's recommendation. The RNA concentration was evaluated by NanoDrop using the A260/A280 ratio. The quality of the extracted RNA was confirmed by 1% agarose gel electrophoresis and observation of the 28S and 18S ribosomal RNA bands. A DNase treatment was performed (Fermentas, 1 *μ*g RNA, 0.5 *μ*l RNA inhibitor, 1 *μ*l DNase1, 2 *μ*l buffer, and 15.5 *μ*l sterile water), and the RNAs were transformed into cDNA using a reverse transcription kit according to the manufacturer's protocol (Takara). Finally, a quantitative SYBR® Green RT-qPCR was performed using a thermal cycler (Applied Biosystems; ABi) in a total volume of 10 *μ*l containing 2.8 *μ*l H_2_O, 1 *μ*l cDNA, 1 *μ*l specific primers, 0.2 *μ*l ROX, and 5 *μ*l SYBR® Green. The CT comparison method (*ΔΔ*CT) was used, and normalization to the GAPDH gene was performed. The primers were designed by Beacon Designer 7 and are shown in Table 1.

### 2.7. Western Blotting

In brief, total proteins were extracted from the testes after homogenization in a lysis buffer [15]. The protein concentration was evaluated by Bradford's test, and 30 *μ*g protein/sample was evaluated by 12% SDS-PAGE and then transferred to polyvinylidene difluoride (PVDF) membranes by wet transfer. The membranes were then blocked (for p-JNK and GPX4, 5% NFDM/TBST; for CHOP, 5% BSA/TBST; and for *β*-actin, 5% skim milk) and incubated overnight at 4°C with the primary antibodies according to the protocols of the respective suppliers and the following dilutions: anti-phospho-JNK monoclonal (1 : 1000), anti GPX4 monoclonal (1 : 200), and anti-CHOP/GADD153 monoclonal (1 : 2000). The membranes were then washed (3x, 15 min at 25°C) in 1x PBS to remove unbound antibodies prior to incubation with the secondary antibodies: anti-goat, anti-rabbit, or anti-mouse IgG antibodies conjugated to HRP (2 h at room temperature). Finally, observation of the protein bands was performed using an enhanced chemiluminescence system (ECL, Santa Cruz, USA) following the manufacturer's instructions. Protein band normalization was conducted using *β*-actin as an internal control.

### 2.8. Statistical and Image Analyses

Statistical analyses were performed using IBM SPSS Statistics 25.0 software, and graphics were designed using GraphPad Prism (version 8.00). Densitometric analyses of the western blots were performed using ImageJ software (version 1.42q). The data were reported as mean ± SEM. One-way ANOVA (Tukey's post hoc test) was used to compare more than two groups (control, VCL, and VCL-DFX groups), and *p* < 0.05 was considered significant.

## 3. Results

### 3.1. Effects of DFX on Body Weight, Testicular Volume, and Testicular Morphometric Parameters in VCL Rats

**Figure 2:**
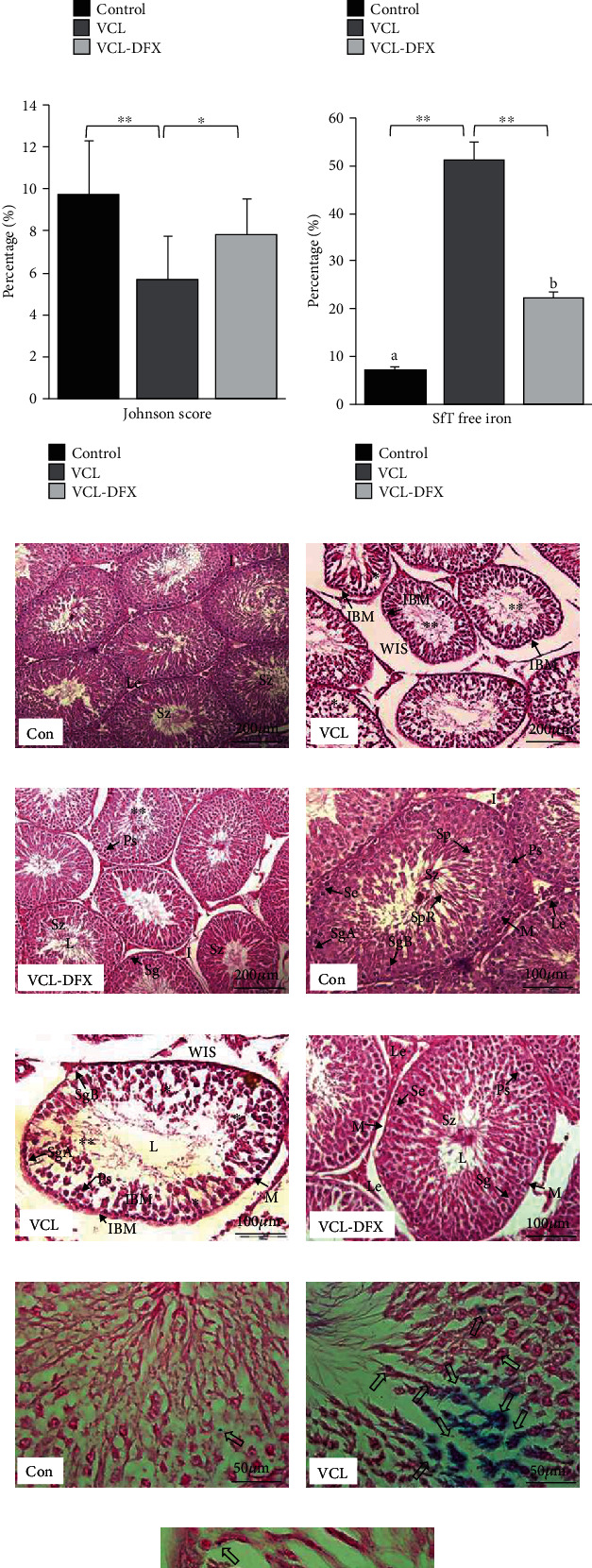
VCL-induced testicular histological damage and free iron content decrease after DFX administration. (a) Mean mitotic index. (b) Mean spermatogenesis index. (c) Mean Johnson score. (d) Mean free iron level from SfTs (seminiferous tubules). (e–g) Representative haematoxylin/eosin-stained testis section (Con = control; VCL and VCL-DFX) at 2 different magnifications: ×20 (e–g) and ×40 (h–j). (k–m) Representative iron accumulation in stained testis sections (Con = control; VCL and VCL-DFX), ×100 magnification. Le: Leydig cells; Se: Sertoli cell; SgA: spermatogonia type A; SgB: spermatogonia type B; Ps: primary spermatocyte; Sp: secondary spermatocyte; I: interstitial space; M: myoid cell; Sz: spermatozoa; WIS: wide interstitial space; IBM: irregular basal membranes; vacuole-like structures and loss of compactness (∗), degraded cells or tubules with minimal spermatozoa content (∗∗), and blue spots show iron accumulation (⇨). Mean ± SEM; ^∗^*p* < 0.05, ^∗∗^*p* < 0.01, and ^∗∗∗^*p* < 0.001; a vs. b (*p* = 0.04) represents significant difference in the three experimental groups.

The average body weight on the day of sacrifice and left testicular volume were compared among the 3 treatment groups. The results showed that unlike the mean body weight, which was similar across the groups (control, 342.89 ± 14.77; VCL, 356.21 ± 7.36; and VCL-DFX, 367.41 ± 7.91 in g), the mean volume of the left testicle was significantly lower in the VCL group than in the control group (in cm^3^, respectively, 1.10 ± 0.12 versus 1.48 ± 0.09; *p* = 0.04). In contrast, this parameter was significantly increased in the VCL-DFX group (1.44 ± 0.07 cm^3^) compared with the VCL group (*p* = 0.04) and approached the value of the control group. When testicular morphometric parameters were evaluated among groups, we observed that the mean mitotic index, percentage of spermatogenesis, and Johnson's score were significantly (*p* < 0.05) lower in the VCL group compared with the control group and that these parameters approached the control group levels (*p* < 0.05) in DFX-treated VCL rats (Figures 2(a)–2(c)). No significant difference was observed between the VCL-DFX group and the control group (*p* > 0.05).

Varicocele-induced histopathological damages to the testes, such as wide interstitial space, increased irregular basement membranes, the presence of vacuole-like structures, increased cell debris in the lumen of the seminiferous tubules, and loss of germ cells, were higher in the VCL group than in the control group. In contrast, these multiple alterations were markedly less pronounced when VCL rats were treated with DFX (Figures 2(e)–2(j)).

### 3.2. Effect of DFX on Testicular Free Iron in VCL Rats

The impact of iron chelation by DFX was evaluated via Perl's staining. As shown in Figures 2(k)–2(m), the VCL group showed an excessive accumulation of iron in the seminiferous tubules. In contrast, the VCL-DFX group showed a significant (*p* < 0.05) decrease in Perl's staining compared with the VCL group (Figure 2(d)).

### 3.3. Effect of DFX on Sperm Parameters in VCL Rats

**Figure 3:**
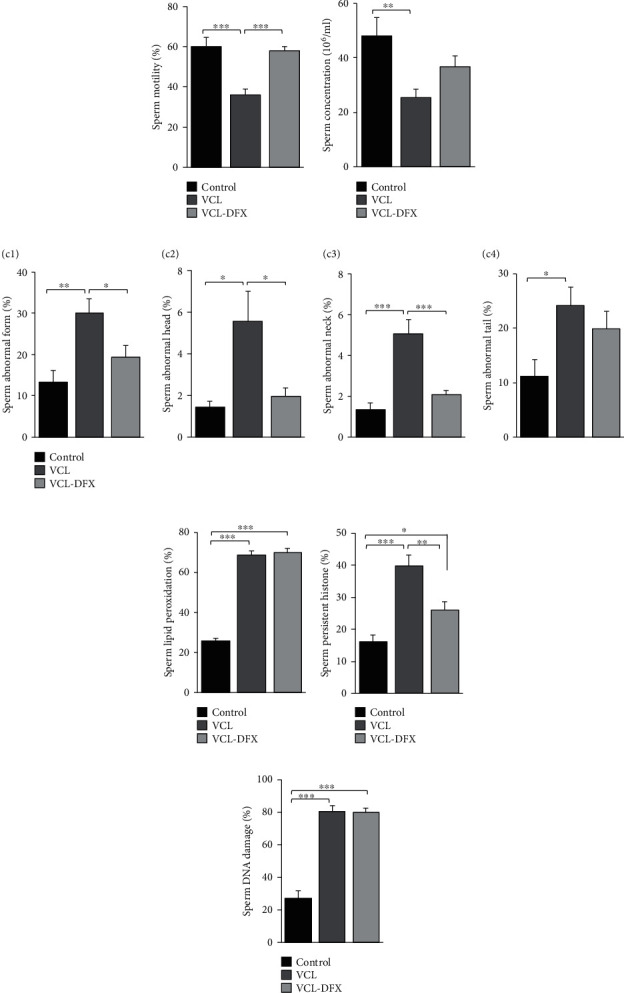
DFX administration improves spermatozoa function and integrity in VCL-induced rats: (a) spermatozoa motility, (b) spermatozoa concentration (10^6^/ml), (c1) spermatozoa abnormal morphology, (c2–c4) detailed spermatozoa abnormal morphology (c2: head, c3: neck, and c4: tail), (d) spermatozoa lipid peroxidation, (e) spermatozoa persistent histone content, and (f) spermatozoa DNA damage. Data are presented as mean ± SEM. ^∗^*p* < 0.05, ^∗∗^*p* < 0.01, and ^∗∗∗^*p* < 0.001 show significant difference between the three experimental groups: control, VCL, and VCL-DFX (VCL rats supplemented with DFX).

As expected from previous work, Figure 3 shows that both sperm motility (Figure 3(a)) and sperm concentration (Figure 3(b)) were significantly lower in the VCL group compared with the control group. Also as expected, the percentage of spermatozoa exhibiting abnormal morphologies was significantly increased in the VCL group compared with the control group (Figure 3(c1)). Administration of DFX to VCL animals (VCL-DFX group) restored motility and decreased the percentage of morphologically abnormal spermatozoa, restoring these parameters to the level of control animals (Figures 3(a) and 3(c1)). In contrast, DFX supplementation did not significantly improve spermatozoa concentration (Figure 3(b)).

It should be noted that a more detailed examination of the different types of sperm morphological abnormalities including abnormal head/neck junctions (any abnormality affecting the site of attachment of the sperm head with the sperm midpiece) and abnormal sperm tails (any abnormalities affecting flagellar structure encompassing the sperm midpiece region containing mitochondria and the flagella) revealed that all abnormal morphologies were increased in the VCL group. Only the percentage of sperm cells showing abnormalities in the tail region was not restored to the control level in the DFX-treated VCL group, whereas all other abnormal morphologies were reduced (Figures 3(c2)–3(c4)).

Other classical VCL-associated spermatozoa alterations were monitored, and in agreement with previously reported data, spermatozoa from VCL animals showed a significant increase in lipid peroxidation (as revealed by the BODIPY C11 test), an increase in DNA/nucleus damage and immaturity as shown by the AO test and higher levels of persistent histones compared with spermatozoa from control animals (Figures 3(d)–3(f)). Treatment with DFX only partially restored the integrity of the sperm nucleus of the VCL animals via a decrease in persistent histone content (Figure 3(e)). Treatment with DFX did not reduce the level of sperm lipid peroxidation or the extent of the DNA damage that was likely associated with the characteristic oxidative stress associated with VCL (Figures 3(d) and 3(f)).

### 3.4. Effect of DFX Effect on the VCL-Induced Testis ER/UPR Response

In order to analyze the effect of DFX on the VCL-induced testis ER/UPR response, we first had to compare the 2-month and 4-month post-VCL induction observations with regard to testicular and spermatozoa parameters. This was necessary because although we had previously reported the ER/UPR status in rat testis 2 months postsurgery [15], we had no information concerning the ER/UPR status 4 months following VCL induction. Since VCL is a chronic condition with progressive oxidative stress leading to cumulative testicular destruction and germ cell apoptosis, we questioned whether the kinetics of the testis ER/UPR response might be different at this later time point.

#### 3.4.1. Evaluation of VCL-Induced Testicular and Spermatozoa Damage 4 Months Post-VCL Induction

**Figure 4:**
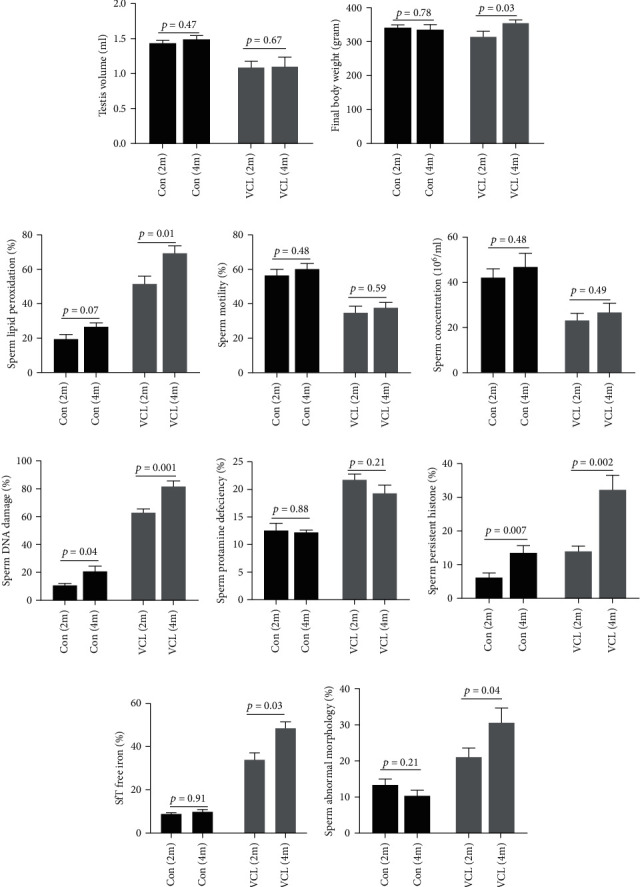
Changes over time for rat anatomical parameters, sperm parameters, and testicular iron content in control and VCL groups. Gross anatomical parameters including (a1) mean final body weight and (a2) mean left testicular volume. Selected semen parameters including (b1) spermatozoa concentration in million/ml, (b2) percentage motile cells, and (b3) percentage of spermatozoa showing lipid peroxidation. Sperm chromatin compaction status with more specifically (c1) percentage of persistent histone, (c2) percentage of protamine-deficient cells, and (c3) percentage of sperm nuclear DNA damage. (d1) Abnormal sperm morphology. (d2) Free iron content of seminiferous tubules (SfTs) in the control (2 *vs*. 4 months) and varicocele (2 *vs*. 4 months) animals. The data represent the mean ± SEM, and an independent *t*-test was performed between two groups. A *p* value < 0.05 is considered significant.

Figure 4 illustrates that 4 months post-VCL induction, gross anatomical parameters in the rat (body weight and testicular volume) were similar to those of 2-month VCL animals. Furthermore, although sperm concentration and motility were similar to those of 2-month VCL animals, sperm lipid peroxidation was significantly increased in animals after 4 months. In addition, Figure 4 shows that the deleterious effect on sperm nuclear integrity was exacerbated after 4 months of VCL as revealed by increased AB staining in the 4-month VCL group when compared with the 2-month VCL group. This is in accord with increased oxidative stress damage provoking nuclear decondensation and is supported by the similar elevated nuclear protamine deficiencies observed for the two time points (Figure 4(c2)).

Of note is that sperm DNA damage in the control animals also showed a significant increase upon aging. Abnormal sperm morphology was also more pronounced in 4-month VCL animals than it was in 2-month VCL animals, whereas this was not observed for age-matched control animals. Finally, testis iron content was significantly higher in 4-month VCL rats than it was in 2-month VCL rats.

#### 3.4.2. Evaluation of the ER/UPR Testicular Response 4 Months following VCL Induction

With regard to ER/UPR status, it appeared that the testis response 4 months post-VCL was strikingly distinct from that observed in 2-month VCL animals. Similar to observations made in 2-month VCL animals, the very first mediator of the ER/UPR stress pathway, the testis chaperone Bip/HSPA5/GRP78, was not elevated in 4-month VCL animals. In contrast to activation of the late ER/UPR phase pathway IRE-1/XBP1s/pJUNK observed in the testis after 2 months of VCL [15], none of the 3 classical ER/UPR pathways were found to be activated in the testis after 4 months of VCL (supplementary figures 1 and 2). Only the mitochondrial proapoptotic signals appeared to be elevated as evidenced by increased accumulation of Bim mRNA and a reduction in Bcl-2 mRNA (supplementary figures 1 and 2). Furthermore, it is interesting to note that testicular accumulation of the antiapoptotic Bcl-2 mRNA was found to be lower 4 months post-VCL induction than it was in age-matched control animals (supplementary figures 1 and 2). However, this was not coupled with increased testicular Bax/Bak mRNA accumulation, nor with increased caspase-3 protein content (supplementary figures 1 and 2). In contrast, Bax and Bak steady-state mRNA levels were lower in the 4-month VCL testis than they were in the 2-month VCL testis. With respect to activation of the testis antioxidant response that accompanies both the VCL and ER/UPR stress responses, we show that there was no difference in Nrf2 mRNA accumulation between controls or VCL animals regardless of the duration of VCL (supplementary figure 2). However, a significant reduction in NRF2 testis protein content was observed in the testis of animals after 4 months post-VCL induction (supplementary figures 1 and 2). NRF2 testis protein content was also reduced in 4-month VCL animals when compared with age-matched control animals (supplementary figures 1 and 2). In agreement with the decreased NRF2 testis protein content, testis GPX4 protein content (the corresponding gene is a well-known target of NRF2 *trans*-acting factor) was also reduced in 4-month VCL animals (supplementary figures 1 and 2).

### 3.5. Does 2 Months of DFX Supplementation Modify the Testis ER/UPR Response in 4-Month VCL Animals?

**Figure 5:**
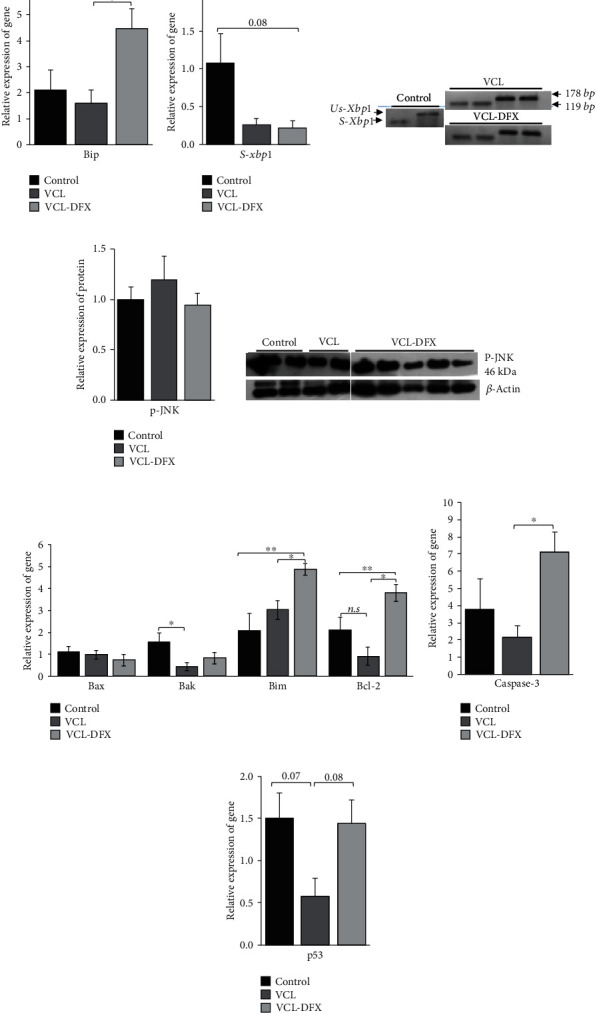
Impacts of DFX chelation on Bip/HspA5/Grp78 transcript and UPR/IRE-1-mediated apoptosis in varicocelized testis: (a) relative accumulation of Bip mRNA; (b1) relative accumulation of s-xbp1 mRNA; (b2) detection of uns-Xbp1 (178 bp band) and s-Xbp1 (119 bp band) mRNA by RT-PCR (2.5% agarose gels); (c1) p-JNK protein content evaluated by western blot (c2); (d) relative mRNA accumulation of mitochondrial-mediated apoptosis markers (Bax, Bak), proapoptosis mediator (Bim), and antiapoptosis mediator (Bcl-2); (e, f) relative mRNA accumulation of caspase-3 and p53 as mediators of programmed cell death. The relative accumulation of mRNA and proteins was normalized to the GAPDH transcript and *β*-actin antibody, respectively. Data are presented as mean ± SEM. ^∗^*p* < 0.05 and ^∗∗^*p* < 0.01, significant difference between study groups: VCL (varicocele), VCL-DFX (varicocelized rats under DFX treatment), and control groups.

**Figure 6:**
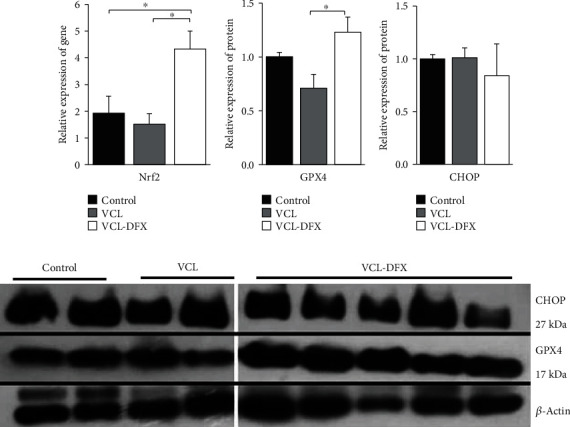
DFX treatment activates the PERK/Nrf2/ARE ER/UPR pathway, but not the PERK/CHOP pathway in the testis of varicocelized rat. (a) Relative accumulation of the Nrf2 transcript. (b) Relative accumulation of the GPX4 protein. (c) Relative accumulation of the CHOP protein. (d) Representative western blots showing the detection of GPX4 and CHOP proteins. The relative accumulation of mRNA and proteins was normalized to the GAPDH transcript and *β*-actin antibody, respectively. Data are shown as mean ± SEM. ^∗^*p* < 0.05 showed significant difference between study groups: VCL (varicocele), VCL-DFX (varicocelized rats under DFX treatment), and control groups.

As shown in Figure 5(a), DFX supplementation for 2 months led to a surprisingly significant increase in testicular Bip/HSPA5/Grp78 mRNA, the first mediator of the ER/UPR response. We next examined the ER/UPR downstream pathways and found that DFX supplementation had no effect on the testicular IRE-1/XBP1s/pJUNK pathway as neither Xbp1s mRNA nor the pJUNK protein *was upregulated* (Figures 5(b) and 5(c)). Similarly, DFX supplementation had no effect on the PERK/CHOP ER/UPR pathway as the CHOP testis content was unchanged in all of the animal groups monitored (Figure 6(c)). However, downstream of these ER/UPR membranous players, we found that DFX supplementation provoked increased accumulation of proapoptotic effector mRNAs such as Bim and Bak and the antiapoptotic effector Bcl-2 (Figure 5(d)). Supplementation with DFX appeared to drive the testis further towards apoptosis as evidenced by the increased steady-state level of caspase-3 mRNA (Figure 5(e)) as well as by the increased accumulation of p53 mRNA (Figure 5(f)).

With regard to the antioxidant response that is a component of the ER/UPR and VCL testis response, we show that DFX supplementation provoked a significant increase in NRF2 mRNA accumulation (Figure 6(a)). The DFX-induced overexpression of NRF2 in the testis was further confirmed by the observation that the GPX4 protein was significantly increased in VCL-DFX testes (Figure 6(b)).

## 4. Discussion

An extensive literature review on the etiology of VCL revealed that ROS play a central role in mediating deleterious effects on the testis and germ cells [1, 22]. Seminiferous tubules are composed of Sertoli cells rich in endoplasmic reticulum and mitochondrial [33] organelles known to store a significant amount of iron [34]. It has been shown that testicular iron storage is increased in VCL [5, 9–11, 24–26]. As excess intracellular iron can become toxic due to its ability to accelerate the production of ROS via the classical Fenton/Haber–Weiss reactions, we tested whether the administration of an iron chelator, DFX, could improve sperm parameters and reduce VCL-induced damage in an animal model of surgically induced VCL.

We show that DFX treatment can decrease testicular free iron overload, protect the testicular tissue from VCL-induced damage, and improve certain sperm parameters (motility and morphology). However, we also show that DFX treatment did not significantly improve spermatozoa concentration measured in cauda epididymis even though the testicular mitotic index and Johnson score were significantly increased. In addition, although DFX treatment significantly decreased the nuclear persistent histone content of VCL spermatozoa, showing an improvement in nuclear quality, this was not accompanied by a decrease in sperm DNA damage.

Of note is that while DFX treatment of VCL animals significantly reduced the percentage of spermatozoa with abnormal morphology, its ameliorative effect was shown to be concentrated on the sperm head and neck, but not on the sperm tail regions. Our initial assumption was that by chelating VCL-mediated testicular iron overload, the iron-induced ROS damage to germ cells could be significantly diminished. This was not fully supported as cauda spermatozoa alterations that are intimately linked to oxidative stress (a high level of lipid peroxidation and nuclear damage) were still observed in VCL-DFX-treated animals. However, it is interesting to note that DFX treatment of VCL animals did succeed in restoring spermatozoa mobility and reducing the proportion of spermatozoa presenting with abnormal head morphology, two parameters that are also closely linked with the impact of oxidative stress on sperm function [35, 36].

One limitation of our study was that based on observations from previously reported treatments, only a single dose of DFX was administered [37, 38]. At this point, we do not know whether other doses could have been more beneficial. Although this awaits additional research, we believe it is rather unlikely because, with the dose administered, we observed beneficial effects (to testicular tissue structures and some functional sperm parameters) in combination with aggravating effects (increased oxidative stress). A question that would merit further investigation is whether or not DFX-chelated iron is removed from the VCL testis. DFX is normally expected to increase renal excretion of iron, but it is possible that due to the specific vascular context of the VCL testis, the removal of chelated iron is limited. If this is indeed the case, it may explain the observed increase in oxidative stress as it has been suggested that chelated iron may contribute to oxidative damage to the same extent as free iron.

Since these conflicting results could be attributed to the dose and duration of DFX treatment administered to the VCL animals used in this study [39–41], we decided to conduct a deeper investigation into the homeostasis of the VCL-DFX-treated testis. We recently reported that the endoplasmic reticulum/unfolded protein response (ER/UPR response), in which oxidative stress is a major player, is likely to be involved in the VCL testis [15]. Therefore, this response was monitored in the testes of the different groups of animals used in the present study. Because we had never evaluated the ER/UPR response of the VCL testis 4 months following surgically induced VCL, it was necessary to monitor the former and compare it to our recent report in which the testis ER/UPR response was analyzed 2 months post-VCL induction [15]. Similar to the observation made 2 months post-VCL induction, the Bip/HSPA5/GRP78 chaperone testis content, the very first mediator of the ER/UPR [13, 15] stress pathway, was not increased at the 4-month time point. In contrast with our previous observation that the late ER/UPR sensor pathway, IRE-1/XBP1s/pJUNK, was activated in the testis after 2 months post-VCL induction, none of the 3 membrane sensor pathways (including PERK, ATF6, and IRE1) were activated in the 4-month VCL testis [15]. Only the mitochondrial-associated apoptotic pathway (as evidenced by increased Bim and decreased Bcl-2 mRNA content), one end point of the ER/UPR pathways, was elevated in the 4-month VCL testis. However, this activation was to a lesser extent when compared with the 2-month VCL testis since neither Bax/Bak nor caspase-3 was found upregulated [15]. Finally, we monitored the testis antioxidant response that accompanies VCL and the ER/UPR response and found that contrary to observations from the 2-month VCL testis [15], the NRF2 protein content and the protein content of one of its known target antioxidant genes (GPX4) were reduced in the 4-month VCL testis. Altogether, these observations suggest that the ER/UPR pathways are no longer activated 4 months after surgically induced VCL. This may be unsurprising as new equilibria are likely to be reached in such a chronic stress-type situation. These observations are consistent with previous studies showing that excess iron and/or oxidative stress not only led to the accumulation of unassembled proteins in the ER but also resulted in reduced Bip/HSPA5/GRP78 expression over time [42–44]. Accordingly, reports of chronic VCL in humans have shown that Bip expression is reduced and is associated with reduced semen quality [45]. Recent studies also revealed an association between compromised sperm maturation and motility with reduced Bip expression in rats in addition to infertile individuals with idiopathic asthenozoospermia [46–49].

In this context, it was surprising to observe that DFX treatment boosted the testis ER/UPR response as evidenced by increased accumulation of the Bip/HSPA5/GRP78 ER chaperone. This event was not associated with any evidence of activation of the downstream ER/UPR membranous sensors (PERK and IRE-1; see Hosseini et al. [15]). However, we recorded a clear increase in apoptotic players (Bim, Bak, caspase-3, and p53) and an increase in the antioxidant response (increased levels of NRF2 mRNA and GPX4 protein in the DFX-treated VCL testis). These data suggest that although DFX was able to limit iron overload-mediated ROS damage to testicular tissue, spermatogenetic function, and sperm cells, this was in parallel with activation of the ER/UPR pathways leading to a proapoptotic response. This provides a likely explanation as to why DFX treatment was unable to restore spermatozoa concentrations to control levels. In addition, it is interesting to note that Bip was immunolocalized to the sperm head and neck compartments, but not to the tail region [48, 49]. This concurs with our observation that DFX treatment, which provoked an increase in Bip content, restored the abnormal head and neck sperm phenotypes, but not the abnormal sperm tail phenotypes. As Bip is involved in protein folding, this may also explain why after DFX stimulation of testicular Bip content we recorded an improved histone to protamine exchange in late differentiating germ cells, a process that requires marked protamine synthesis and ER maturation events [50]. We should point out that our observations contradict earlier reports, which have shown that DFX decreases cellular apoptosis via the inhibition of P-JNK signaling and mitochondrial-dependent cell death [20, 51].

Moreover, our data suggest that DFX treatment provoked a testis antioxidant response as evidenced by the upregulation of NRF2 and one of its targets, GPX4, the stability of which was shown to be associated with the ability of the latter to bind Bip [52]. It is difficult to ascertain whether this antioxidant response is secondary to the activation of the ER/UPR stress pathway PERK [53] or the result of DFX-induced oxidative stress. Since there was no evidence that the PERK/CHOP pathway was triggered, because neither PERK nor CHOP was upregulated in the DFX-treated testis samples, we made the assumption that we had observed DFX-associated oxidative stress. Regardless of the origin, this oxidative stress may explain the increased level of spermatozoa lipid peroxidation and nuclear damage recorded in the DFX-treated VCL animals. This may also explain the inability of DFX to diminish abnormal sperm tail morphology that could originate from oxidative damage to this sperm mitochondrial-associated compartment. Nevertheless, this could contradict with the observation that DFX treatment improved spermatozoa motility in 4-month VCL animals. However, abnormal sperm midpiece organization may be unrelated to mitochondrial energy efficiency. DFX may help to remove iron overload from sperm mitochondria, which has been associated with low ATP generation and alteration of sperm motility as well as with increased ROS generation [4, 54–58]. It is of interest to note that in agreement with our observations, Perera et al. reported that an increase in sperm DNA damage was recorded upon initiation of iron chelation therapy in *β*-thalassemic patients with iron overload [59].

In conclusion, DFX treatment of surgically induced VCL rats provokes a dual response marked by an increase in testicular proapoptotic markers (Bim, caspase-3, and p53) associated with an increase of antiapoptotic and antioxidant markers (Bcl-2, NRF2, GPX4, and Bip). This is in agreement with previous reports that have shown iron chelation leads to increased p53 expression acting as the “guardian of the genome” together with increased caspase-3 expression in order to restore cell homeostasis [60, 61]. It remains to be determined whether the antioxidant response is mediated by the ER/UPR stress pathways or inherent to DFX. Altogether, the data reported herein show that the iron chelation therapeutic strategy partially restored VCL-induced damage to testicular tissue and sperm function. It will be interesting to test whether coadministration of antioxidants with iron chelators could provide added benefit to the treatment of VCL. This may be possible as we and others recently reported that antioxidant administration could partially improve VCL tissue and cell alterations in animal models as well as in humans [25, 62, 63]. Such treatment combinations (i.e., iron chelation+antioxidant) have shown therapeutic value in other disease situations [64–66].

## Figures and Tables

**Table 1:** List of primers used for real-time RT-PCR analysis.

Gene	Primer sequence (5′-3′)	GenBank no.	Amplicon size (bp)
*Grp78*	*F TAACAATCAAGGTCTACGAAGG* *R CCATTCACATCTATCTCAAAGGT*	NM_013083.2	193
*Splice xbp-1*	*F CTGAGTCCGCAGCAGG* *R CTTGTCCAGAATGCCCAAAAGG*	NM_001271731.1	119
*Unspliced xbp-1*	*F GTCCGCAGCACTCAGACTAC* *R CTGGGGAAGGACATTTGAAAAAC*	NM_001004210.2	178
*Nrf2*	*F TGCCATTAGTCAGTCGCTC* *R GTGCCTTCAGTGTGCTTC*	NM_031789.2	99
*Bcl-2*	*F ACTTCTCTCGTCGCTACCGTC* *R AAGAGTTCCTCCACCACCGT*	NM_016993.1	106
*Bax*	*F GGATCGAGCAGAGAGGATGG* *R ACACTCGCTCAGCTTCTTGG*	NM_017059.2	91
*Bak*	*F CAGAGAGGTGGTTGGGTGG* *R GTGGGTTGGGGAGAGGTTTAG*	NM_053812.1	181
*Bim*	*F CACAAACCCCAAGTCCTCC* *R AGTCTCATTGAACTCGTCTCC*	NM_171988.2	152
*Caspase-3*	*F CGGTATTGAGACAGACAGTGGAAC* *R GCGGTAGAGTAAGCATACAGGAAG*	NM_012922.2	90
*p53*	*F CCGACTATACCACTATCCACTAC* *R CACAAACACGAACCTCAAAGC*	NM_030989.3	147
*GAPDH*	*F TGCCGCCTGGAGAAACC* *R TGAAGTCGCAGGAGACAACC*	NM_017008.4	121

## Data Availability

Data are available on request.

## Abbreviations

VCL: Varicocele
ER: Endoplasmic reticulum
UPR: Unfolded protein response
PERK: Protein kinase R- (PKR-) like endoplasmic reticulum kinase
ATF6: Activating transcription factor 6
IRE1: Inositol-requiring enzyme 1
DFX: Deferasirox
GRP78: 78 kDa glucose-regulated protein
XBP1: X-box binding protein 1
p-JNK: Phosphorylated JNK
CHOP: C/EBP homologous protein
GPX4: Glutathione peroxidase 4
Nrf2: Nuclear factor erythroid 2-related factor 2
PBS: Phosphate-buffered saline.

## Supplementary Materials

Supplementary Materials Supplementary Figure S1: comparison of time-dependent changes in relative levels of left testicular mRNA/protein content of players involved in the mitochondria and ER/UPR survival/death pathways in control and VCL animals. A1–A4: relative mRNA abundance of markers of the mitochondria proapoptotic (Bax, Bim, and Bak) and antiapoptotic (Bcl-2) responses. B1, B3: relative mRNA abundance of markers of the ER/UPR response (Bip/Grp78 and PERK/Nrf2). B2: relative caspase-3 mRNA abundance. C1–C4: relative CHOP protein content, p-JNK, NRF2, and GPX4 markers. In each analysis, 2-month *vs*. 4-month VCL animals were compared with control animals. The data are presented as mean ± SEM, and an independent *t-*test was performed between two groups. A *p* value < 0.05 is considered significant. Supplementary Figure S2: in a surgically induced varicocele model in rats, it was shown that the testis ER/UPR stress pathways were triggered as a result of the hyperthermia, hypoxia, and oxidative stress generated [15]. Among the three main membrane sensors of ER stress that can be mobilized in ER/UPR responses, we had previously shown that after 2 months of VCL, the late pathway IRE1/XBP1s/pJNK was the most prominent, itself triggering the expected proapoptotic and prooxidative responses [15], as revealed by the increased expression of NRF2 and caspase-3. After 4 months of VCL, the testicular response was different, with no involvement of any of the 3 ER membrane sensors. Only a proapoptotic response was revealed, as suggested by the decrease in the antiapoptotic factor Bcl-2 and the increase in the proapoptotic factor Bim.
