# Supplementary material for: Deferasirox, an Iron-Chelating Agent, Improves Testicular Morphometric and Sperm Functional Parameters in a Rat Model of Varicocele
Source: Oxid Med Cell Longev. 2021 Apr 8;2021:6698482. doi: 10.1155/2021/6698482 (PMC8052147; doi:10.1155/2021/6698482)
Supplement: Supplementary Materials — Supplementary Figure S1: comparison of time-dependent changes in relative levels of left testicular mRNA/protein content of players involved in the mitochondria and ER/UPR survival/death pathways in control and VCL animals. A1–A4: relative mRNA abundance of markers of the mitochondria proapoptotic (Bax, Bim, and Bak) and antiapoptotic (Bcl-2) responses. B1, B3: relative mRNA abundance of markers of the ER/UPR response (Bip/Grp78 and PERK/Nrf2). B2: relative caspase-3 mRNA abundance. C1–C4: relative CHOP protein content, p-JNK, NRF2, and GPX4 markers. In each analysis, 2-month vs. 4-month VCL animals were compared with control animals. The data are presented as mean ± SEM, and an independent t-test was performed between two groups. A p value < 0.05 is considered significant. Supplementary Figure S2: in a surgically induced varicocele model in rats, it was shown that the testis ER/UPR stress pathways were triggered as a result of the hyperthermia, hypoxia, and oxidative stress generated [15]. Among the three main membrane sensors of ER stress that can be mobilized in ER/UPR responses, we had previously shown that after 2 months of VCL, the late pathway IRE1/XBP1s/pJNK was the most prominent, itself triggering the expected proapoptotic and prooxidative responses [15], as revealed by the increased expression of NRF2 and caspase-3. After 4 months of VCL, the testicular response was different, with no involvement of any of the 3 ER membrane sensors. Only a proapoptotic response was revealed, as suggested by the decrease in the antiapoptotic factor Bcl-2 and the increase in the proapoptotic factor Bim. [file 6698482.f1.docx]

**VCL**

HYPERTHERMIA HYPOXIA OXIDATIVE STRESS

**ER/UPR STRESS**

**CASPASE** **3**

*APOPTOSIS*

PERK

ATF4

CHOP

ATF6

CHOP

IRE-1

XBP1s

pJNK

**NRF2**

**BIM**

**BAX**

**BAK**

**BCL2**

**VCL 2 months VCL 4 months**

**PERK/CHOP -- --**

**IRE1/XBP1s/pJNK**

**NRF2 --**

**CASPASE 3 --**

**BIM --**

**BCL2 --**

**BIP/GRP78/HSPA5**

***Antiapoptotic***

***Proapoptotic***

**Figure S2**


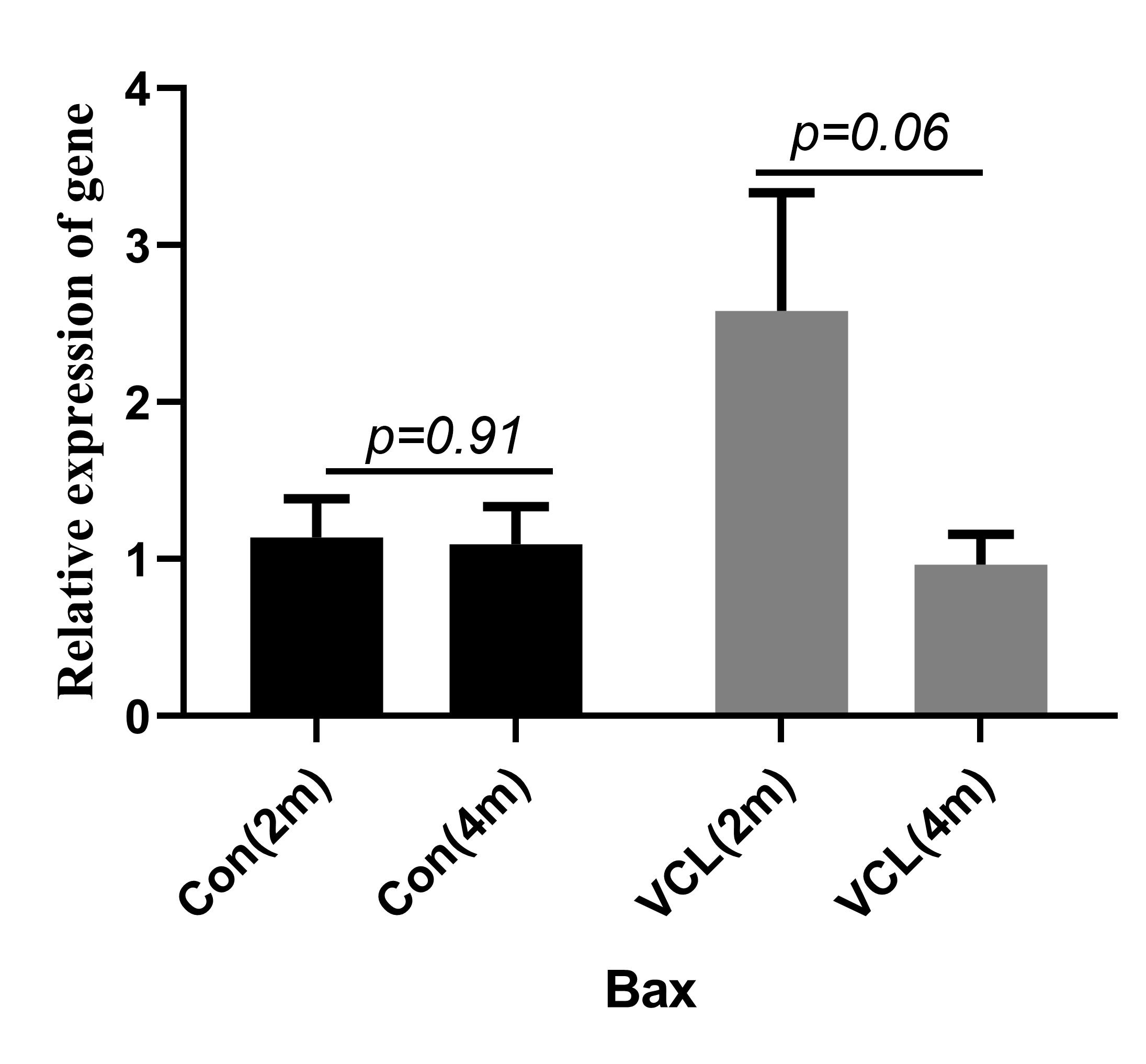


**Figure S1.**

**A1 A2**

**A3 A4**

**B1 B2 B3**

**C1 C2**

**C3 C4**
